# Reaction kinetics and properties of MDI base poly (urethane-isocyanurate) network polymers

**DOI:** 10.1080/15685551.2021.1971858

**Published:** 2021-08-26

**Authors:** Juan Li, Shengling Jiang, Liang Ding, Lingfang Wang

**Affiliations:** a Department of Polymer and Composite Material, School of Materials Science and Engineering, Yancheng Institute of Technology, Yancheng, China; b Key Laboratory of Carbon Fiber and Functional Polymers of Ministry of Education, College of Materials Science and Engineering, Beijing University of Chemical Technology, Beijing, China

**Keywords:** Poly (urethane-isocyanurate), trimerization, step heating

## Abstract

Since the trimerization of isocyanate occurs easily and controllably to form a clear trifunctional isocyanate ring, this reaction is an ideal candidate for the synthesis of a clear poly(urethane-isocyanurate) network polymer. Poly(urethane-isocyanurate) network polymer (PUI) was prepared from diphenylmethane diisocyanate (MDI) and propylene glycol (PPG) by cyclotrimerization of isocyanate group (NCO). It was proved that the expected product was successfully prepared by NCO determination, fourier transform infrared (FTIR) and gel permeation chromatography (GPC) characterization. The mechanical and thermal properties were characterized. Through the effects of catalyst dosage, polyurethane prepolymer molecular weight, reaction time, reaction temperature and MDI addition on the reaction process, it is determined that under certain other conditions, the step heating method is better for cyclotrimerization reaction. Generally, the better heating conditions are 60 °C/1 h + 80 °C/4 h + 100 °C/2 h + 120 °C/2 h + 140 °C/2 h + 160 °C/2 h. The results of thermogravimetric analysis (TGA) and mechanical properties showed that with the increase of cross-linking points in the polymer structure, the thermal stability, tensile strength, tensile modulus and hardness of PUI increased, while the elongation at break decreased significantly. The glass transition temperature (Tg) of PUI is around 45 °C, and it can be seen that the elastic modulus of the material can range from 58 to 1980 MPa. X-ray diffraction results show that the rubber phase represented by the flexible segment and the plastic phase represented by the rigid structure are amorphous.

## 1. Introduction

In polyurethane chemistry, a well-known network formation method is the trimerization of isocyanates [1]. In this reaction, three isocyanate groups undergo a step-by-step addition reaction to form isocyanate ring, which is usually catalyzed by strong nucleophilic reagent (Nu−) [2]. Since the trimerization of isocyanate occurs easily and controllably to form a clear trifunctional isocyanate ring, this reaction is an ideal candidate for the synthesis of a clear polymer network [3–5]. The trimer reaction of isocyanate can be used to prepare many kinds of materials, such as resin [6], foams [7], aerogel [8,9], hydrogel [10,11], polyurethane gradient material [12,13], shape memory material [14], etc. and it has been applied to aerospace, aviation, biology, medicine, shipbuilding and other fields.

In the past decades, the research on the trimerization of isocyanates has been continued, including the effects of catalysts, isocyanate types, reaction time reaction, temperature, and so on.Askadskii [3,5,15,16] prepared polyurethane isocyanurate by two-step method, using TDI\PPG as raw material, undergoing trimerization reaction fastly at a temperature higher than 100 °C, and then completing the crosslinking reaction at 160–180 °C. Goleneva et al. [17] studied the kinetics of 2,4-toluene diisocyanate (TDI) interacting with tetrahydrofuran-propylene oxide copolymer to form polymer with polyurethane isocyanate structure in the presence of selective catalyst by infrared spectroscopy. The formation conditions of reticular polyurethane isocyanurate polymer were defined. A one-step synthesis method of polyurethane isocyanurate is proposed, which can replace the traditional two-step method to prepare gradient materials based on poly(urethane–diisocyanurate). Korshak et al. [18] discussed in detail the formation mechanism, catalyst selection, reaction temperature and time of TDI-Based Poly (urethane-isocyanurate) network, and considered that the formation of isocyanurate ring can be regarded as the result of the attack of C atom and N atom on isocyanurate ring in isocyanate trimer after PGE epoxy ring is opened, The best reaction condition is to use bisphenol A diglycidyl ether as catalyst and heat the reaction at 177–230 °C for 8–10 h. Dekamin et al. [19] used TDI and poly(propylene glycol) as raw materials to prepare polyurethane isocyanurate network by prepolymer method in the presence of tetrabutylammonium phthalimide-N-oxyl (TBAPINO) or tetraethylammonium 2-(carbamoyl)benzoate (TEACB) as effective and metal-free cyclotrimerization catalysts. Based on the above reaction mechanism, Askadskii et al. [12,13] prepared complex amine catalyst with N, N-Dimethylbenzylamine (DMBA) and epoxy resin(ED-22), which was used as the catalyst for cyclotrimerization to synthesize poly (epoxy-isocyanurate) network. Due to the presence of complex amine catalyst, the cyclotrimerization reaction is selective and the side reactions are reduced.

On the basis of previous work, poly(urethane–diisocyanurate) was prepared by two-step method in this work. Polyurethane prepolymer (PUP) was prepared from polypropylene glycol (PPG) and diphenylmethane diisocyanate (MDI), then cyclic trimerization was carried out between pup and expected proportion MDI under the condition of complex amine catalyst, and finally poly (urethane-isocyanurate) network polymer (PUI) was prepared. The cyclic trimerization process was studied by FTIR tracking method, and the mechanical properties (tensile strength, elongation at break) and thermal properties (TG/DTG) of PUI were tested.

## 2. Experimental

### 2.1. Material

Bis(4-isocyanatophenyl)methane (MDI-50) was purchased from Wanhua Chemical Group Co. Ltd. Propylene glycol (Mn = 1050, 2100, 3260) were purchased from Shandong Bluestar Dongda Co. Ltd. N, N-Dimethylbenzylamine (DMBA), bisphenol A diglycidyl ether were purchased from Acros. Dibutyltin dilaurate (DBTDL), acetone, dibutylaminer were commercially obtained. PPG needs vacuum dehydration before use, and molecular sieve is used to remove water before reagent use.

### 2.2. Instrumentation and methods

NCO content was determined by di-n-butylamine titration. The theoretical value of NCO% is calculated according to the formula ($NCO\%=\frac{\begin{array}{c} (m(HDI)/M(HDI)-\\ m(PPG)/M(PPG))*42.02 \end{array}}{m(HDI)+m(PPG)}*100\%$).

Molecular weights (*M*_w_ and *M*_n_) and polydispersity indexes (PDI, *M*_w_/*M*_n_) of the polymers were estimated in THF by a Waters gel permeation chromatography (GPC) system(Waters, 515 HPLC Pump).

Fourier transform infrared (FTIR) spectra measurements were performed using a Nicolet Nexus 670 (FTIR) instrument (USA). The spectra obtained at resolution 8 cm^−1^ in the range 4000 ~ 400 cm^−1^. Samples were prepared with attenuated total reflection ATR.

The reaction kinetics is studied by infrared tracking method. The theoretical basis of this analysis method is absorbance, which is commonly used in infrared quantitative analysis. The absorbance is directly proportional to the concentration, and it is additive. In the infrared spectrum, CH_3_ corresponding to 2972 cm^−1^ and 2869 cm^−1^ does not participate in the cyclic trimerization reaction, which can be regarded as the reference peak, and the cyclic trimerization reaction process must be accompanied by the change of – NCO content. Therefore, the asymmetric stretching vibration peak of NCO group at 2270 cm^−1^ continues to weaken. Therefore, the infrared spectrum measured in the process of cyclotrimerization can be used to analyze the change of NCO group content in the process of reaction. The absorbance A = A2270/A2869, and the change of NCO base content can be tracked by the change of A_i_ (relative absorption density measured at a certain time point) and compared with A_0_ (initial relative absorption density). At a given temperature, once the A_i_/A_0_ curve tends to equilibrium (which indicates that the reaction speed is slowed and the interaction is inhibited), the temperature is increased by 20 °C. Therefore, the reaction temperature rises in steps, and the tracking stops when the infrared characteristic peak corresponding to NCO group disappears.

Thermogravimetric analysis (TGA) was performed on a Perkin–Elmer TGS-2 under nitrogen ambience with a heating rate of 10°C/min in the temperature range of 25 ~ 800°C.

X-ray diffraction analysis (XRD) was performed on Brooke D8-advance with the scanning rate of 10°/min.

Tensile test of dumbbell specimens was carried out on a testing machine (CMT4104, Shenzhen SANS Testing Machine, Shenzhen, Guangdong Province, China) at a velocity of 500 mm/min, according to ASTM D882.

### 2.3. Synthesis of poly(urethane–isocyanurate) (PUI) network polymer

**Scheme 1.**
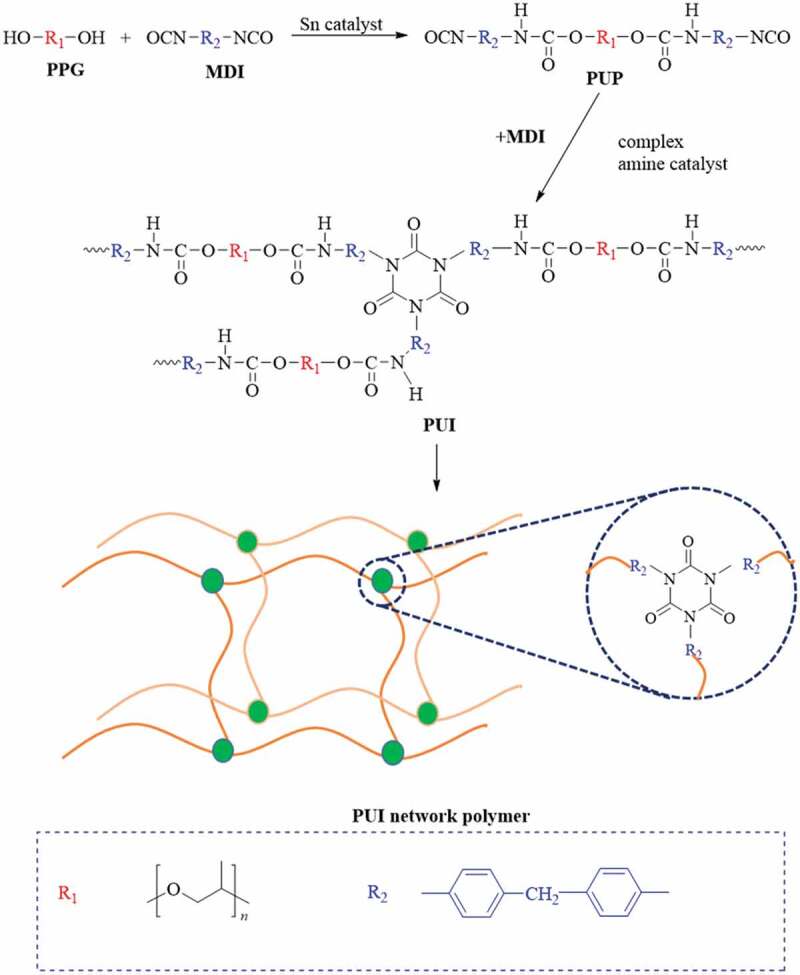
Synthesis route of poly(urethane–isocyanurate)(PUI) network polymer

The synthetic route of poly(urethane–isocyanurate) (PUI) network polymer prepared by two-step method is shown in Scheme 1. The first step is the traditional urethane formation reaction, that is, under certain conditions, oligo diol (PPG) reacts with diisocyanate (MDI) according to R = 2 to obtain NCO terminated polyurethane prepolymer (PUP). In the second step, the cyclic trimerization of PUP was carried out in the presence of complex amine catalyst, and the curing was carried out by step heating method. Finally, the network polymer based on poly (urethane-isocyanurate) was obtained.

The typical synthesis process is as follows: (1) oligo diol (PPG1000, 105 g, 0.1 mol) was added into a 500 ml four port flask with thermometer and stirrer, dehydrated at 105 °C under vacuum for 2 h, and cooled to 65 °C. Bis(4-isocyanatophenyl)methane (MDI,50.4,0.2 mol) was added, reacted for 30 min, raised the temperature to 80 °C, continue the reaction for 1 h, and then catalyst dibutyltin dilaurate (DBTDL) was added. The NCO value was tested every 1 h until the NCO value was constant, then the reaction stopped, and the product was vacuumed until there was no obvious bubble in the system, and the PUP was sealed and stored for standby. (2) PUP (60 g) was added into a 500 ml flask, and the system temperature was controlled at 45–55 °C. Then MDI(18 g) was added into the reaction system, and the complex amine catalyst (1:20 mass ratio of N, n-dimethylbenzylamine (DMBA) and diglycidyl ether of bisphenol A (DGEBA)) was rapidly prepared. The catalyst was slowly dropped into flask and mixed evenly. The system was stirred at 50 °C under vacuumed for 15 min. Then the mixture was poured into a preheated mold and placed in a vacuum drying oven for bulk polymerization under vacuum. The step heating conditions were 60°C/1 h + 80°C/4 h + 100°C/2 h + 120°C/2 h + 140°C/2 h + 160°C/2 h. Finally, poly (urethane-isocyanurate) network polymer was obtained.

## 3. Results and discussion

### 3.1. Effects on synthetic poly (urethane-isocyanurate) network

#### 3.1.1. The amount of catalyst and the molecular weight of PUP

**Figure 1.**
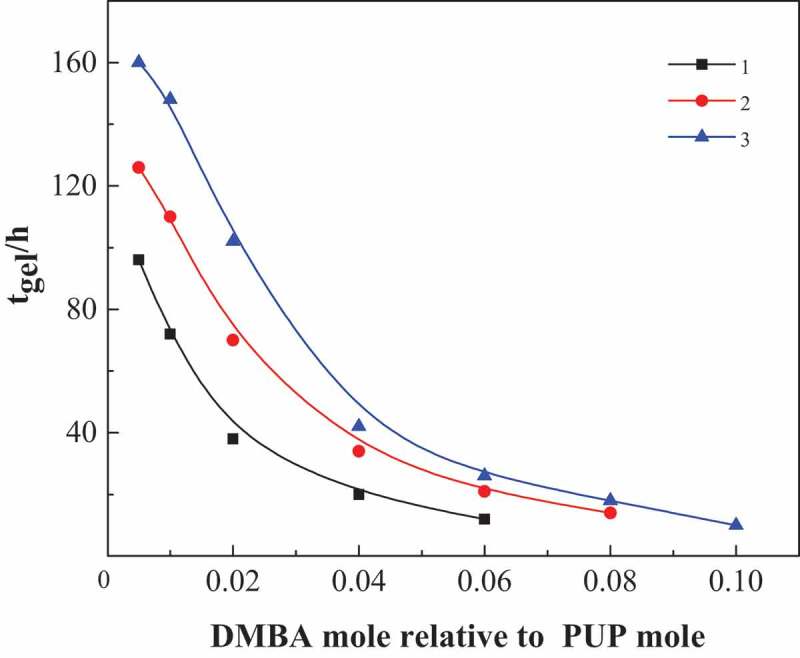
The influence of time of curing time on DMBA concentration for PUP(sample NO. 1, 2, 3 from Table 1)

The curing process is the process of cyclic trimerization and continuous formation of isocyanurate ring. Because the prepared prepolymer is an oligomer terminated by NCO group, it can be regarded as an oligomer diisocyanate. It can carry out bulk ring trimerization under the action of catalyst without adding MDI. Because the content of – NCO in the system is low and the reaction speed is relatively slow, the influence of catalyst dosage on curing time can be measured more accurately. Therefore, in order to simplify the curing process, a series of prepolymer pups are synthesized with PPG and MDI as raw materials, as shown in Table 1. It can be seen from the NCO% and GPC results in Table 1 that PUPs with different molecular weights were synthesized by PPG and MDI with different molecular weights. Then, complex amine catalyst was added and the cyclic trimerization curing reaction was carried out at 80 °C. The relationship between the concentration of N, N- two methyl benzyl amine (DMBA) and gel time is shown in Figure 1.

**Table 1.** Synthesis of NCO-functionalized prepolymers (PUP)

NO.	oligomer diol	diisocyanate	Mn	Mw	Mw/Mn	NCO%^a^
1	PPG1000	MDI	2600	3600	1.40	3.48%
2	PPG2000	MDI	4500	6900	1.52	3.36%
3	PPG3000	MDI	7300	10,700	1.47	3.46%

^a^The theoretical value of NCO% is 3.43%.

As shown in Figure 1, the curing rate of poly (urethane-isocyanurate) network polymer is related to the amount of catalyst used and the molecular weight of the prepolymer itself. With the increase of DMBA concentration, the reaction rate was significantly accelerated and the complete curing time was significantly reduced. If the catalyst is excessive, the curing reaction will be uncontrolled due to the obvious exothermic reaction of the system. Therefore, the molar ratio of DMBA to prepolymer in the catalyst is more appropriate between 0.04 and 0.06. At the same time, with the increase of PUP molecular weight, the curing time under the condition of the same amount of catalyst increases greatly, that is, under the condition of a certain amount of catalyst, the curing time of PUP prepared by PPG1000 is shorter. Therefore, the following experiments do not make special instructions, and PPG1000 is selected as oligomer glycol.

#### 3.1.2. Reaction time and temperature

**Figure 2.**
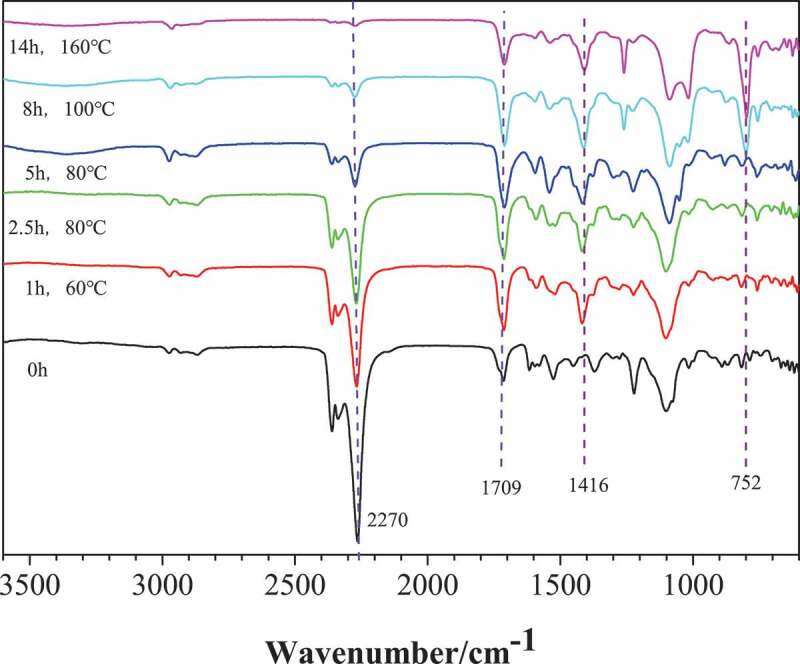
FTIR of the polymer network under different reaction time and reaction temperature

The infrared spectrum of PUI network polymer produced by MDI\ PPG1000 as raw material and two-step method at different reaction time and reaction temperature is shown in Figure 2. It can be seen from Figure 2 that when the reaction time is 0 min, the mixed system of PUP and MDI has no peaks at 1416 cm^−1^ and 752 cm^−1^, and there is a strong NCO stretching vibration absorption peak at 2270 cm^−1^. After adding the catalyst and curing at 60 °C for 1 h, the characteristic peaks corresponding to isocyanurate ring appear at 1416 cm^−1^ and 752 cm^−1^, and the peak intensity at 2270 cm^−1^ begins to weaken, which proves that the ring trimerization reaction has begun. The presence of – NCO at 2270 cm^−1^ indicates that the reaction is not complete. Similarly, the same conclusion can be obtained by analyzing the spectrum when the curing temperature is 80 °C and the curing time is 2.5 h. When the curing temperature was 160 °C and the curing time was 14 h, the NCO group basically disappeared, and the peaks at 1709 cm^−1^, 1416 cm^−1^ and 752 cm^−1^ became stronger. It shows that NCO groups react completely to form a large number of isocyanurate rings, and poly (urethane-isocyanurate) network polymers are obtained.

#### 3.1.3. Amount of MDI

**Figure 3.**
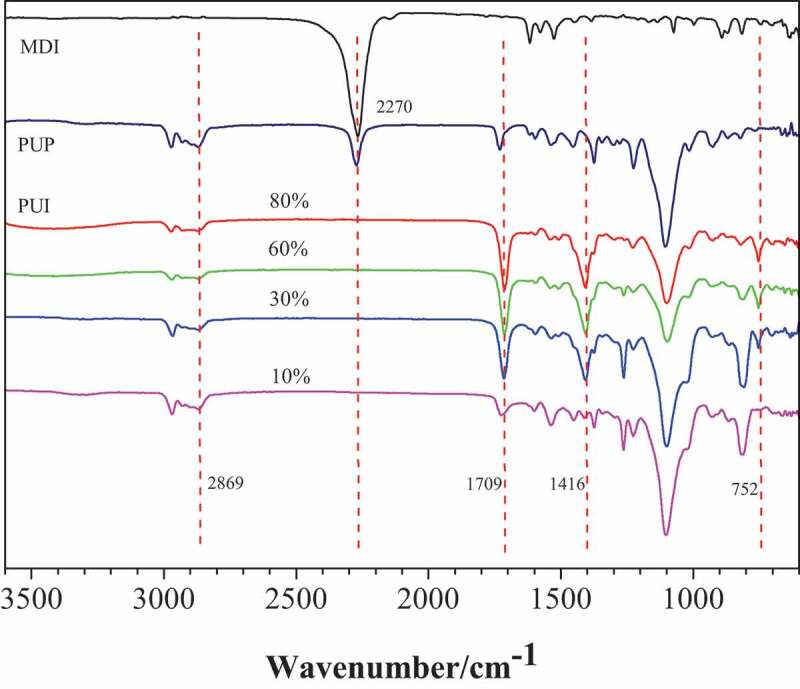
FTIR of the PUI generated by different amount of MDI. Different curve representatives to different mass fraction of MDI(relative to PUP): 10%, 30%, 60%, 80%

Under certain other conditions, the reaction process was characterized by FTIR by changing the addition amount of MDI in the second step. The infrared spectrum of PUI prepared by trimerization of PUP and MDI with different mass fractions is shown in Figure 3.

As shown in Figure 3, with the increase of MDI content, the intensity of infrared peaks (1709 cm^−1^, 1416 cm^−1^ and 752 cm^−1^) corresponding to isocyanurate ring gradually increases, indicating that the concentration of isocyanurate ring in cross-linked polymer gradually increases. This shows that in the process of preparing poly (urethane-isocyanurate) network polymer, the higher the mass fraction of diisocyanate added (compared with PUP), the more the proportion of ring structure in PUI. In this way, we can change the proportion of flexible structure (rubber component) and rigid structure (isocyanurate ring as crosslinking point) in the resulting polymer network.

**Figure 4.**
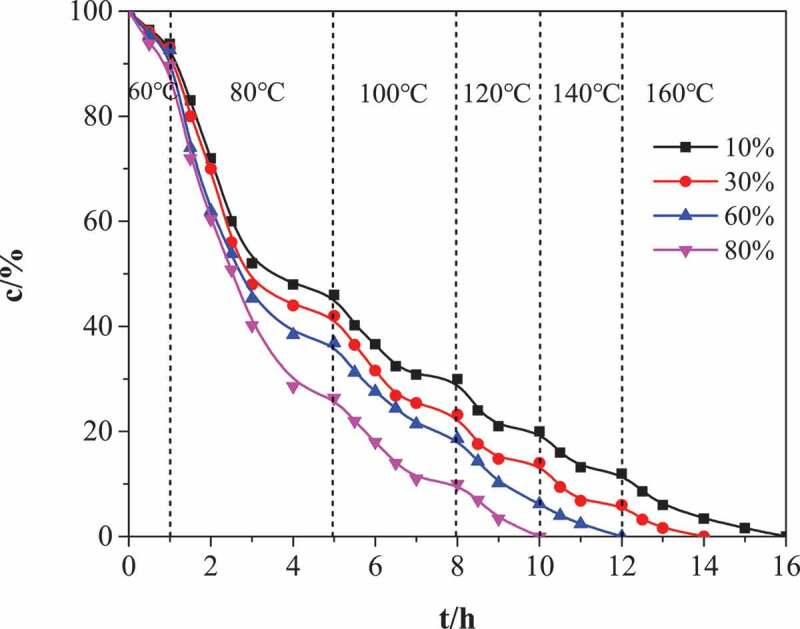
The change of isocyanate group content (*c*) in the process of cyclotrimerization with MDI and PUP. Different curve representatives to different mass fraction of MDI(relative to PUP): 10%, 30%, 60%, 80%

The relative change of NCO (A2270) relative to CH_3_ (A2869) infrared absorption peak during cyclotrimerization is tracked by FTIR. The results are shown in Figure 4. The initial temperature of ring trimer reaction is selected at 60 °C to control gel time and side reaction. If the initial set curing temperature is too high, the curing speed of the reaction system may be too fast in the presence of diisocyanate and catalyst. On the one hand, the local temperature will be too high and side reactions will occur. On the other hand, the acetone and bubbles in the system will not be completely removed, resulting in foaming inside the cured product and flat surface. After 1 hours of reaction, the gel initially formed and granular material appeared in the system. Then heating up to 80 °C, the reaction was most intense (from 80 °C to gel forming), and then the reaction rate decreased. This is because more isocyanurate rings are formed in bulk polymerization, resulting in a rigid polymer structure, which hinders the diffusion and reaction of reaction groups. Since the flexible structure (rubber component) in the polymer network almost completely reacts when the temperature reaches 100–120 °C, while the rigid structure (isocyanurate ring as the cross-linking point) can fully react at 140–160 °C. Therefore, in order to realize the complete conversion of NCO, the final reaction temperature must be increased. It can be found by infrared that the isocyanurate ring will not be formed quantitatively when the temperature reaches 160 °C.

As shown in Figure 4, the cyclic trimerization reaction rate is the highest in the low content MDI system. When the content of MDI in the system increases, the consumption rate of NCO group decreases and the reaction rate slows down, that is, the conversion rate decreases under the same catalyst and temperature conditions. This is because with the increase of the proportion of hard rigid isocyanate rings in poly (urethane-isocyanurate) network polymers, the glass transition temperature of the rigid structure of the generated network polymers gradually increases, approaches the reaction temperature, the mobility of macromolecular segments decreases, and the possibility of contacting reaction groups decreases. In conclusion, the reaction temperature of cyclotrimerization reaction is better in the way of step heating. Generally, the better heating conditions are 60°C/1 h + 80°C/4 h + 100°C/2 h + 120°C/2 h + 140°C/2 h + 160°C/2 h。

### 3.2. Properties of poly (urethane-isocyanurate) network polymers

According to Scheme 1, PUP is synthesized from PPG1000 and MDI, and then MDI and amine complex catalyst are added to prepare a series of PUI by step heating (60°C/1 h + 80°C/4 h + 100°C/2 h + 120°C/2 h + 140°C/2 h + 160°C/2 h), as shown in Table 2.

**Table 2.** Synthesis of poly(urethane–isocyanurate) (PUI) network polymers

PUI NO	Content of diisocyanate(wt%)	Shore hardness	Tensile strength(MPa)	Elongation at break(%)	Elastic tensile modulus (MPa)
1	10	84A	9.96	131.91	58.74
2	20	92A	14.66	77.14	110.69
3	30	97A	18.53	43.94	230.26
4	40	69D	23.12	11.06	380
5	60	82D	30.82	3.65	654
6	80	87D	39.28	1.38	1024
7	100	96D	50.06	0.31	1980

^a^Content of diisocyanate(wt%) = m(MDI)/m(PUP)*100%.

#### 3.2.1. Mechanical property

It can be seen from Table 2 that using oligomer diols with the same molecular weight, with the increase of diisocyanate content, the hardness, tensile strength and tensile elastic modulus of the prepared poly (urethane-isocyanurate) network polymer increase correspondingly, while the elongation at break decreases significantly; Among them, the variation range of elastic modulus is wide, from 58 MPa to 1980 MPa.

This shows that the mechanical properties of the polymer depend on the chemical structure of the polymer network, which is a typical poly (urethane-isocyanurate) polymer. Changing the amount of isocyanate in the reaction system can change the structure of the polymer network and the ratio of flexible structure (rubber component) and rigid structure (isocyanurate ring as cross-linking point and its connected aromatic ring) in the resulting polymer network, so as to regulate the mechanical properties of polymer materials in a large range. Macroscopically, such polymers change from soft to hard, that is, from soft rubber to hard rubber, and from soft plastic to hard plastic.

#### 3.2.2. Thermal properties

**Figure 5.**
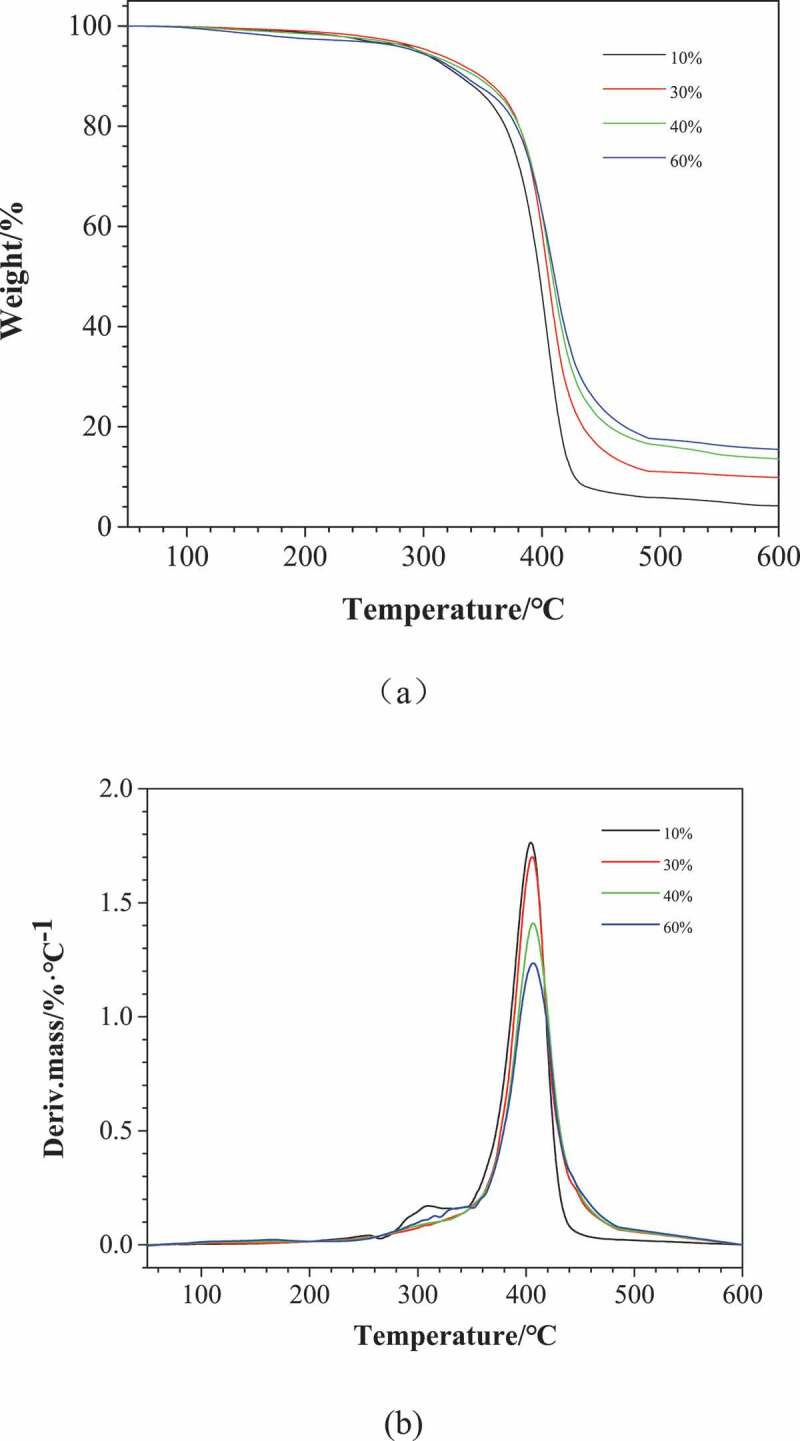
TGA (a) and DTG(b) curves of PUI under N_2_. (10%, 30%, 40% and 60%, respectively corresponding to sample NO.1, 3, 4 and 5 in Table 2)

The thermal weight loss behavior of poly (urethane-isocyanurate) network polymers synthesized with different MDI concentrations is shown in Figure 5. The initial thermal decomposition temperature (T_d5_), weight loss temperature of 50% (T_d50_), maximum thermal decomposition temperature (T_m_) and carbon residue rate of each sample are shown in Table 3. It can be seen from Figure 5 and Table 3 that the initial thermal decomposition temperature of each sample is basically the same, but from the DTG diagram, it can be seen that PUI (10%) has a small thermal weight loss peak at 308 °C, while other curves do not have this weight loss peak. The reason may be that PUI (10%) contains less cross-linked ring structure and flexible short chain occupies the dominant position in the molecular structure. This weight loss peak may be caused by the breaking of urethane bond on flexible short chain and the thermal decomposition of non-crosslinked low molecular substances. PUI has a very obvious thermal weight loss phenomenon between 360 and 450 °C. From the DTG diagram, it can be seen that PUI has an obvious weight loss peak at about 405 °C. The thermal weight loss at this stage can be regarded as the thermal decomposition of isocyanurate ring in the crosslinking point. In short, with the increase of MDI concentration, T_d50_ and T_m_ increased accordingly, and the carbon residue rate at 600 °C also increased. At the same time, the weight loss peak moved slightly to high temperature, and the strength of weight loss peak decreased. This is because the phenyl substituted isocyanurate ring mainly appears in the form of C = O-N-R structure, and there is no unstable H in the ring structure, which makes it have certain heat resistance. In addition, the N atom in the isocyanurate ring structure makes the prepared PUI have certain flame retardancy. Therefore, the thermal stability of PUI increases with the increase of large volume rigid intersection points in the structure.

**Table 3.** Thermal properties of poly(urethane–isocyanurate) (PUI) network polymers

NO.	Content of diisocyanate(wt%)^a^	T_d5_(°C)	T_d50_(°C)	T_m_(°C)	char yield(%)^b^	T_g_(°C)
1	10%	296	398	404	3.82	−46.5
2	30%	300	405	412	8.84	−43.8
3	40%	298	409	412	13.58	−45.6
4	60%	298	411	415	15.43	−44.8

^a^Content of diisocyanate(wt%) = m(MDI)/m(PUP)*100%. ^b^Residual carbon rate at 600 °C.

**Figure 6.**
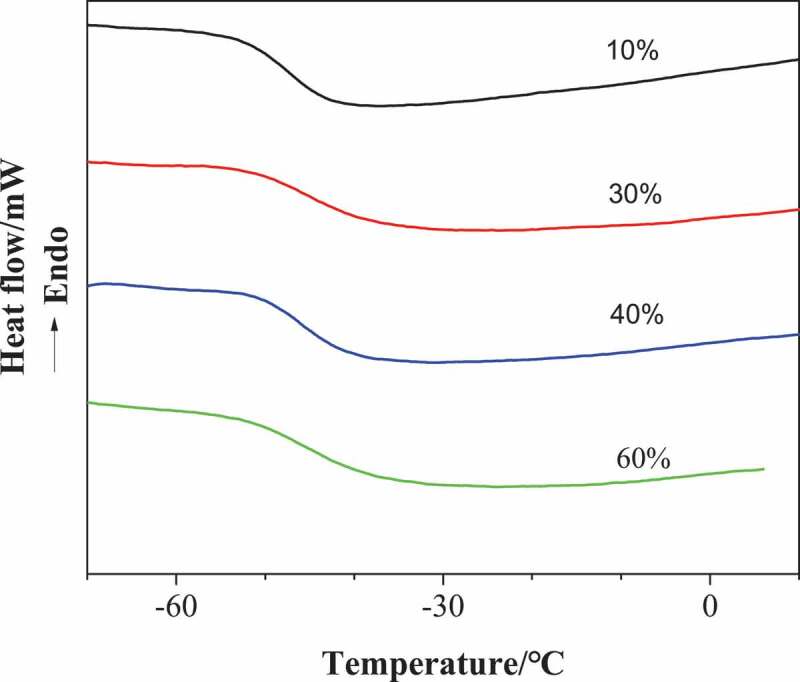
DSC curves of the PUI(10%, 30%, 40% and 60%, respectively corresponding to sample NO.1, 3, 4 and 5 in Table 2)

Figure 6 shows the DSC curves of poly (urethane-isocyanurate) network polymers synthesized with different MDI concentrations. The flexible chain glass transition temperature Tg of the sample is listed in Table 3. It can be seen from the table that the Tg of each sample is about 45 °C and does not increase due to the increase of ring structure in the polymer. This is due to the great difference in surface energy between flexible chain and rigid intersection point. The existence of rigid isocyanurate network will not affect the Tg of rubber phase corresponding to flexible chain.

#### 3.2.3. Crystallinity

**Figure 7.**
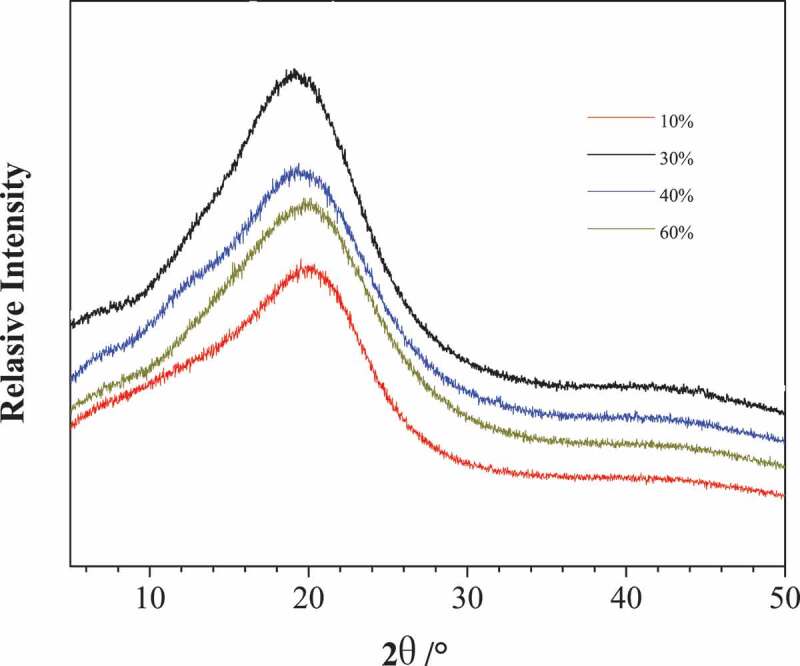
The XRD curve of PUI(10%, 30%, 40% and 60%, respectively corresponding to sample NO.1, 3, 4 and 5 in Table 2)

In order to further characterize the morphology of soft segment and rigid structure in poly (urethane-isocyanurate) network polymer, some PUI network polymers prepared by different MDI content were characterized by XRD, and the obtained spectrum is shown in Figure 7. It can be seen from the figure that there is only a single diffusion peak in all curves, which proves that the rubber phase represented by the flexible segment and the plastic phase represented by the rigid structure (isocyanurate ring and its aromatic ring) are amorphous and amorphous blocks, indicating that the prepared products do not show crystallization. There are two main reasons for this phenomenon. On the one hand, the flexible short chain is composed of the reaction of polypropylene glycol and diisocyanate, and its molecular chain interaction is weak and easy to be randomly arranged. At the same time, the structure of polypropylene glycol contains side group CH_3_, and the structure is irregular, so the crystallinity of flexible chain is low. On the other hand, there are large volume cross-linking points in the obtained network polymer. The existence of ring structure reduces the regularity of plastic phase and shows random winding state. There are many hydrogen bond groups (urea group, urea formate group, etc.) in the material. The force between different kinds of hydrogen bond groups is different. These two factors lead to the difficulty of crystal structure in the product structure.

## 4. Conclusions

Poly (urethane-isocyanurate) network polymer (PUI) were synthesized from MDI and PPG by two-step method. The expected products were successfully prepared by NCO determination, FTIR and GPC. Through the effects of catalyst dosage, pup molecular weight, reaction time, reaction temperature and MDI addition on the reaction process, it is determined that under certain other conditions, the step heating method is better for cyclotrimerization reaction. Generally, the better heating conditions are 60°C/1 h + 80°C/4 h + 100°C/2 h + 120°C/2 h + 140°C/2 h + 160°C/2 h.

The results of performance characterization showed that with the increase of MDI content and the increase of cross-linking point in the polymer structure, the thermal stability, tensile strength, tensile modulus and hardness of PUI increased, while the elongation at break decreased significantly, and Tg basically not changed. The variation range of elastic modulus is wide, from 58 MPa to 1980 MPa. XRD results show that the rubber phase represented by the flexible segment and the plastic phase represented by the rigid structure (isocyanurate ring and its aromatic ring) are amorphous.
